# Diamond Patterns: Cumulative Cornsweet Effects and Motion-Induced Brightening

**DOI:** 10.1177/2041669518770690

**Published:** 2018-07-12

**Authors:** Patrick Cavanagh, Stuart Anstis

**Affiliations:** Department of Psychology, Glendon College, Toronto, ON, Canada; Department of Psychological and Brain Sciences, Dartmouth College, Hanover, NH, USA; Department of Psychology, University of California, San Diego, La Jolla, CA, USA

**Keywords:** adaptation/constancy, lightness/brightness, motion, perception

## Abstract

A Cornsweet edge creates the perception of a step in surface lightness between two adjacent regions of identical mean luminance due to a gradient on both sides. We might imagine that in a concatenated set of these gradients, the lightness steps would accumulate, but they do not. However, a diamond pattern, with each diamond filled with an identical luminance gradient does give a cumulative Cornsweet effect. Here, we offer an illumination explanation for why the cumulative effect is visible in the diamonds but not in the basic ramp grating and we demonstrate that when the diamonds drift, they produce a strong brightening effect (depending on the direction of the motion) and a dimming aftereffect. These effects are consistent with the local luminance gradients and not with the global lightness shift of the cumulative Cornsweet effect.

Figure 1 shows that the concatenation of rectangles with identical luminance gradients does not produce cumulative lightness steps whereas the concatenation of diamonds does (Figure 2). We propose that the diamond shapes support a decomposition into a reflectance step and an illumination gradient. Thus, Figure 3 starts with a set of spatially uniform diamonds that increase in mean reflectance from left to right. Under an illuminant that gets progressively *darker*, the combination of reflectance steps and illumination gradient can give all the diamonds the same mean luminance with an identical luminance gradient within each – the diamond stimulus.

**Figure 1.**
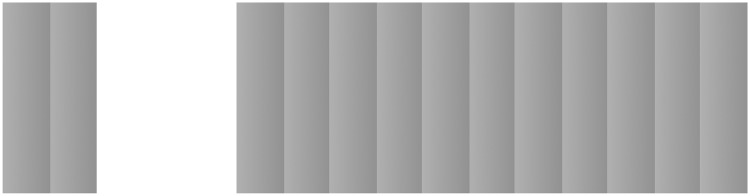
Left: Cornsweet effect. When two rectangles, each with the same light to dark gradient, abut, there appears to be a uniform lightness step from one to the other (Cornsweet, 1970). Right: Several of these rectangles are concatenated to form a sawtooth luminance profile. Here, the lightness steps do not accumulate beyond the first step.

**Figure 2.**
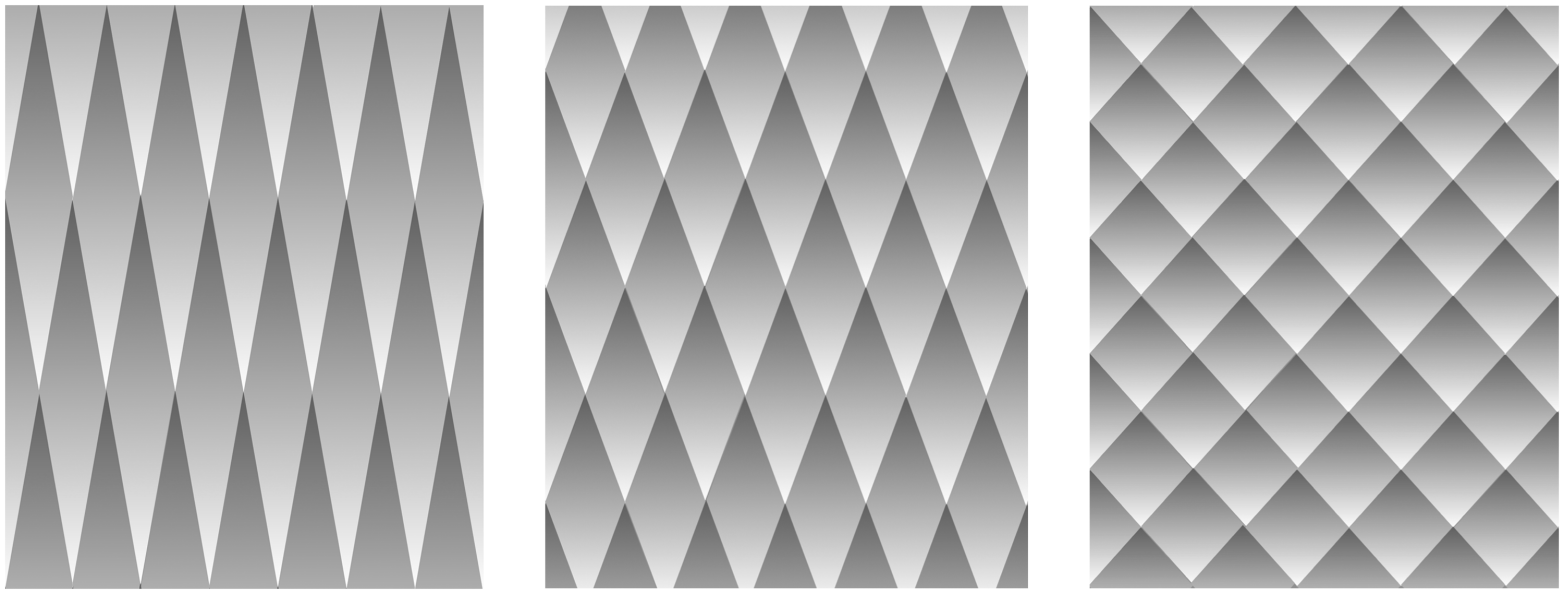
Cumulative Cornsweet effect. Each individual diamond on the left appears to have fairly uniform lightness which then increases from darker for the bottom diamonds to lighter for the upper ones (Watanabe, Cavanagh, & Anstis, 1995). This global lightness shift is opposite in direction to the actual luminance gradients within each diamond. In addition, the cumulative Cornsweet effect is more evident on the left for the pointy diamonds than on the right for the squat diamonds.

**Figure 3.**
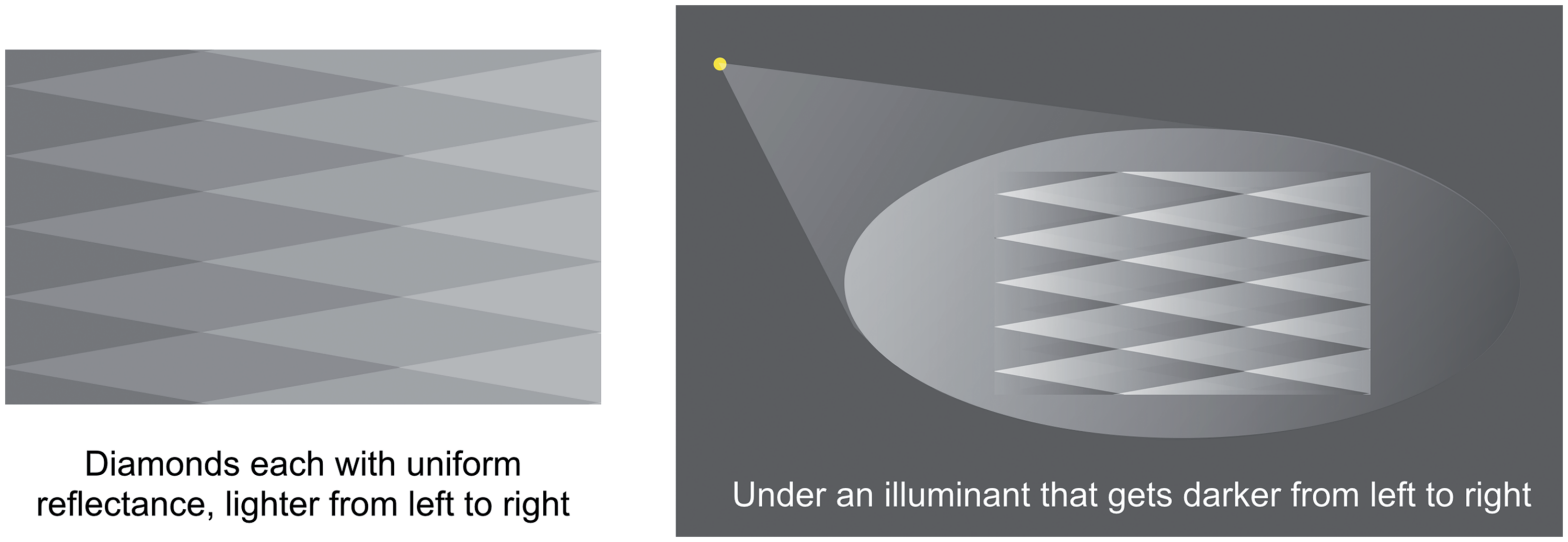
The diamond stimulus can be seen as a combination of a set of diamonds with uniform reflectance (left image), stepping up in reflectance from left to right, viewed under an illumination gradient that gets darker from left to right (right image). The result, on the right, can be a set of identical diamonds with the same mean luminance and the same internal gradients. The visual system then decomposes this into uniform reflectances increasing from left to right seen under a gradient of illumination.

We propose that the critical factor that generates the cumulative effect is the continuous luminance change that occurs *along* each edge in the diamond pattern but not the ramp grating. In this case, the luminance decreases on both sides of the edge, mimicking (although not exactly) the changes that would happen to a fixed reflectance edge under an illumination gradient (see also Figure 4). In contrast, in the ramp grating, the luminance step is constant all along the edge, giving no additional weight to an illumination explanation. Although both the ramp grating (Figure 1) and the diamonds (Figure 2) are equally well modeled as the sum of stepped reflectances and an illumination gradient, there is less evidence for the illumination gradient in the ramp grating, tipping the balance against the decomposition.

**Figure 4.**
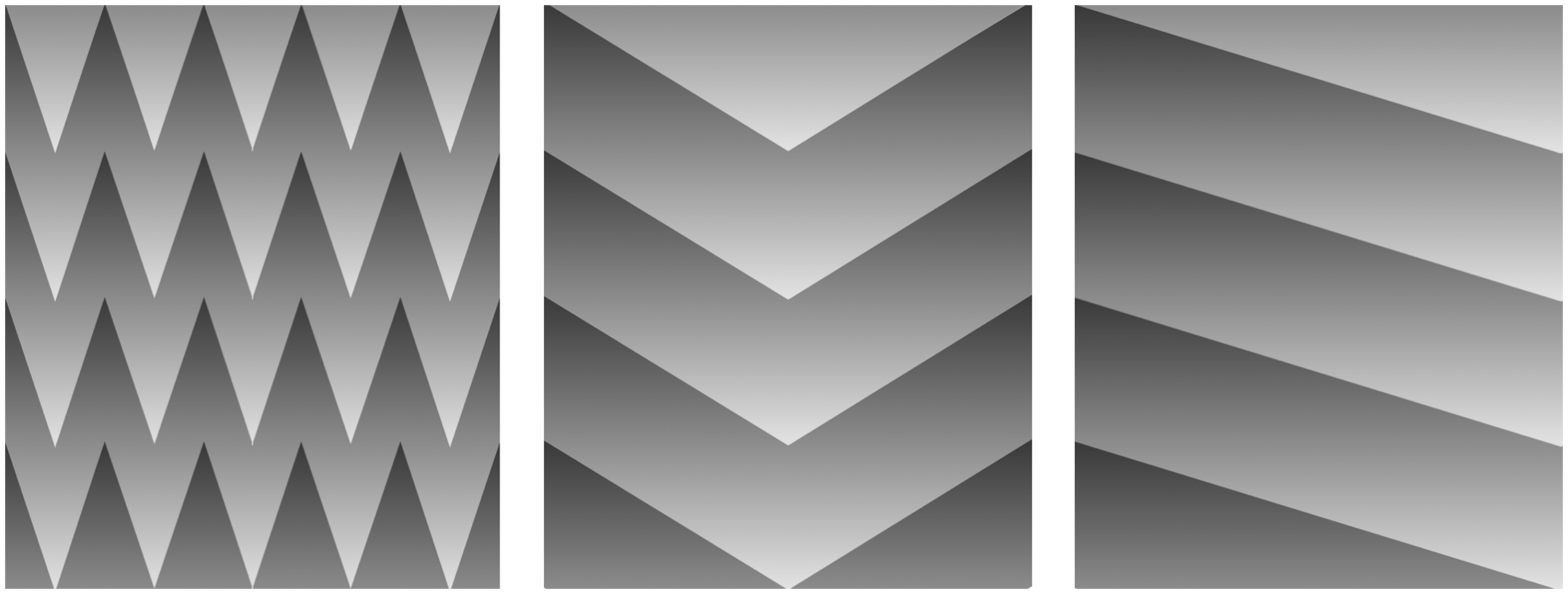
Left: A set of identical spiky bars each having the same luminance gradient also produces a cumulative lightness increase as the mean luminance across the borders increases along the border, as it does in the diamond pattern. Middle: As the angle of the spikes becomes shallower, the cumulative effect weakens. Right: It disappears when the angles are flat as in a ramp grating in Figure 1.

The diamond stimulus also reveals the relative roles of the two luminance gradients – the cumulative, global lightness change between diamonds and the *opposite*, local gradient within each diamond, in producing motion-induced brightening effects.We have previously shown that drifting ramp gratings produce strong brightening and darkening effects (Cavanagh & Anstis, 1986) and that adaptation to such temporal brightness ramps produces dramatic brightening aftereffects (Anstis, 1967, 1979; Anstis & Harris, 1987; Arnold & Anstis, 1993). We now show that the moving diamond patterns produce the same effect as the moving ramp grating and give similar aftereffects (Movie 1). Since these effects are in the same direction as those for the ramp stimulus that has no global gradient, we conclude that the local luminance gradients in the diamonds drive the brightening effects even though the gradient has been perceptually suppressed.

**Movie 1.** Part 1: The two fields move in opposite directions. Every second, they reverse direction, and the motion-induced brightening switches sides. Part 2: Here, when the motion reverses, the direction of the gradients are also reversed to keep the lightening and darkening effects on the same side throughout. After 24 seconds of adaptation, the movie stops and the darkening and brightening aftereffects are visible in the outlined regions.

In conclusion, we suggest that the diamond stimulus and other equivalent stimuli (Figure 4) produce cumulative Cornsweet effects because they strongly support an illumination gradient interpretation. They do so because the luminance changes along the edges make the paired gradients on both sides mimic the luminance difference across a fixed reflectance edge under a luminance gradient. This holds even though the luminances in the diamond stimulus differ by a constant amount rather than by a constant ratio as they should for an illumination effect. In contrast, the edges in a ramp grating have one fixed luminance difference along them and give simply one datum point in favour of an illumination gradient. Finally, both motion-induced brightness effects were consistent with the direction of the internal gradients of the diamonds despite the perceptual reduction of the gradient. This shows that the actual luminance gradient generates the effect and that the perceived lightness gradient does not contribute to motion-induced brightness effects.
